# Effects of Dietary Puffed Jujube Powder on Growth Performance, Apparent Digestibility, and Meat Quality of Hainan Black Goats

**DOI:** 10.3390/ani15152306

**Published:** 2025-08-06

**Authors:** Yi Zhang, Jianzhi Shi, Jiapeng Wang, Keke Li, Xianzheng Qiao, Dong Chen, Tingting Dong, Yuanxiao Li, Yushu Zhang, Renlong Lv

**Affiliations:** 1 College of Animal Science and Technology, Henan University of Science and Technology, Luoyang 471000, China; shijianzhi0427@163.com (J.S.); 241513160129@stu.haust.edu.cn (J.W.); 241413010117@stu.haust.edu.cn (K.L.); 15083368119@163.com (X.Q.); chendong0621@163.com (D.C.); 18586290044@163.com (T.D.); lyx8023@yeah.net (Y.L.); 2 Department of Agriculture, Shinshu University, Matsumoto 390-8621, Japan; zhangys12306@gmail.com; 3 Tropical Crops Genetic Resources Institute, Chinese Academy of Tropical Agricultural Sciences, Haikou 571101, China

**Keywords:** puffed jujube powder, Hainan black goats, growth performance, antioxidant capacity, meat quality

## Abstract

This study was conducted to investigate the effects of puffed jujube powder (PJP) supplementation in the diet on the slaughter characteristics, growth performance, meat quality, and serum antioxidant capacity of Hainan Black (HB) goats. Twenty-four healthy male HB goats, three months old with an initial body weight of 15.12 ± 3.67 kg, were randomly divided into three groups: the 10% PJP group (basal diet plus 10% PJP); the 20% PJP group (basal diet plus 20% PJP); and the control group (basal diet only). After a 10-day adaptation period, a feeding trial was carried out for 90 days in an ad libitum diet environment. The results show that the final body weight of the 20% PJP group was markedly higher (*p* < 0.05) than that of the control group (22.58 ± 0.94 kg vs. 20.45 ± 1.01 kg). The average daily gain of the 20% PJP group was 83.44 ± 1.78 g/d, which was substantially greater (*p* < 0.05) than the 59.22 ± 2.13 g/d of the control group. The feed intake of the 20% PJP group was 713.10 ± 4.54 g/d, notably higher (*p* < 0.05) than the 498.20 ± 4.33 g/d of the control group. In terms of slaughter characteristics, the carcass weight of the 20% PJP group was 13.99 ± 1.22 kg, considerably heavier (*p* < 0.05) than the 11.79 ± 1.38 kg of the control group. The muscle weight of the 20% PJP group was 11.43 ± 1.42 kg, distinctly greater (*p* < 0.05) than the 9.59 ± 1.99 kg of the control group. The slaughter rate of the 20% PJP group was 42.41%, showing a notable increase (*p* < 0.05) compared with the 37.42% of the control group, and the net meat rate of the 20% PJP group was 34.65%, with a significant rise (*p* < 0.05) compared with the 30.43% of the control group. Regarding serum antioxidant capacity and meat quality, the activities of serum antioxidant enzymes, superoxide dismutase (SOD) and catalase (CAT), were conspicuously increased (*p* < 0.05) in the 20% PJP group. The meat shear force of the 20% PJP group was decreased by 12.9%, and the cooking loss was improved by 8.9% in comparison with the control group. In conclusion, the supplementation of 20% PJP in the diet was demonstrated to enhance the growth performance, improve the meat quality, and boost the antioxidant status of HB goats, thus presenting a feasible strategy for optimizing tropical goat production systems.

## Simple Summary

Hainan Black goats are confronted with challenges, including slow growth rates, low meat production, and inferior meat quality under hot and humid conditions. This study aimed to investigate whether supplementing with puffed jujube powder in their diet could enhance growth performance and meat quality. We utilized 24 young male goats, divided into three groups: a control group without puffed jujube powder supplementation, a group supplemented with 10% puffed jujube powder, and a group supplemented with 20% puffed jujube powder. Following a 90-day experimental period, the group receiving 20% puffed jujube powder supplementation exhibited significantly higher growth rates, greater weight gain, and increased feed intake compared with the control group. Additionally, this group demonstrated heavier slaughter weight, enhanced meat yield, and elevated antioxidant capacity in blood serum. Moreover, the meat from this group displayed superior tenderness and reduced cooking loss. In conclusion, supplementing with 20% puffed jujube powder significantly improves growth performance, meat production, and meat quality in Hainan Black goats, providing an effective strategy to enhance goat farming efficiency through utilization of jujube byproducts, thereby offering economic benefits to farmers and promoting environmental sustainability.

## 1. Introduction

The Hainan Black (HB) goat, originating from the Leizhou breed on Hainan Island, China, is renowned for its remarkable disease resistance and thermotolerance [1]. Its meat, characterized by superior quality with uniform fat distribution, mild flavor, and palatability, is favored by consumers [1]. However, small-scale farming predominated within the HB goat production system. Large-scale breeding operations confronted substantial challenges, including low feed utilization efficiency arising from unbalanced ration formulations and impaired disease resistance due to non-standardized management protocols [1]. As a specialized meat-producing breed, the HB goat is also constrained by intrinsic limitations, such as small body size, slow growth rate, and low meat production efficiency [2]. Feed shortages and nutritional deficiencies are further exacerbated by seasonal variations and climatic conditions, which adversely affect production performance [3]. Moreover, under conditions of elevated temperature and humidity, oxidative stress is markedly intensified in HB goats, which induces lipid oxidation and concomitant deterioration of muscle tissue quality, consequently compromising nutritional value [3]. Our prior investigations have demonstrated that dietary supplementation with antioxidants, including α-lipoic acid, significantly improves average daily gain (ADG), meat quality, antioxidant capacity, and feed conversion efficiency in HB goats. This intervention also markedly reduces drip loss and shear force while simultaneously enhancing meat tenderness [2]. Moreover, appropriate supplementation with antioxidants, particularly those derived from natural phytogenic sources possessing dual medicinal and edible functions, has been demonstrated to enhance both antioxidant capacity and immunomodulatory responses in lambs [4,5].

The red jujube (*Ziziphus jujuba* Mill.), widely grown in China and recognized as an important medicinal and edible fruit, produces considerable agricultural residues from the rejection of non-compliant fruits [6,7]. Puffing technology, a process involving high-temperature, high-pressure extrusion followed by sudden decompression, transforms jujube byproducts into nutrient-dense puffed jujube powder (PJP). This technology significantly enhances storage stability through moisture reduction and microbial inhibition while ensuring the retention of key bioactive compounds [7]. Furthermore, PJP effectively retains key nutrients and antioxidants—including polysaccharides, flavonoids, triterpenic acids, polyphenols, and ascorbic acid—which are essential for preserving the jujube powder’s nutritional value and biological activity [6,7,8]. In livestock production, PJP exhibits considerable potential for application. Dietary supplementation with PJP in cattle has been demonstrated to significantly increase average daily gain, enhance antioxidant capacity and immune function, and provide effective prevention against diarrhea [8]. In goat farming, the dietary inclusion of PJP significantly improved growth performance, enhanced serum antioxidant capacity, and elevated immunoglobulin levels [9]. Furthermore, partial substitution of corn with PJP meal in the basal diets of white cashmere goats resulted in elevated serum cholesterol and triglyceride levels, enhanced antioxidant activity, and demonstrated a significant elevation in immunoglobulin G concentrations [10]. Collectively, these findings demonstrate that PJP exhibits considerable potential to enhance meat production performance, reduce feed conversion ratios, improve production efficiency, and facilitate sustainable, health-oriented animal husbandry practices. Although the substantial effects of PJP have been well documented in ruminant nutrition and feeding trials, its implementation in the HB goat breed, a breed distinguished by unique challenges, including slow growth rates, small body size, and susceptibility to oxidative damage induced by heat stress in tropical environments, remains uninvestigated.

Growth performance, carcass characteristics, and meat quality constitute critical indicators for evaluating production efficiency and product quality in the caprine industry [11,12]. Growth performance metrics include body conformation index, average daily feed intake (ADFI), and average daily gain (ADG), reflecting physical development, feed efficiency, and growth rate [13]. Carcass traits, including yield and lean meat versus adipose tissue distribution, are key benchmarks for assessing market value and competitiveness in the meat industry, directly influencing marketability and consumer acceptance of meat quality attributes [14,15]. Analysis of these indicators is critical for animal health and welfare, product quality and feeding techniques, market competitiveness, and sustainable livestock production [16,17]. In view of the above, this study used the above evaluation indexes to investigate the effects of PJP on HB goat slaughter characteristics, growth performance, meat quality, and serum antioxidant capacity, aiming to provide a scientific basis for the addition of PJP in the HB goat production system.

## 2. Materials and Methods

### 2.1. Ethical Treatment

The experimental protocol received approval from the Institutional Animal Care and Use Committee of Henan University of Science and Technology (Approval No. HAUST-2023-017) and the Animal Ethics Committee of the Chinese Academy of Tropical Agricultural Sciences (Approval No. CATAS-2023-79). All procedures were conducted in strict accordance with institutional animal ethics guidelines.

### 2.2. Animals and Diets

The trial was conducted at the Danzhou Experimental Station of the Chinese Academy of Tropical Agricultural Sciences (located in Danzhou, Hainan, China) from April to July 2023. Twenty-four healthy male HB goats, aged 3 months with an initial body weight of 15.12 ± 3.67 kg and uniform physiological status, were purchased from Hainan Benfu Agricultural Technology Co., Ltd. (Haikou, China). The puffed jujube powder (PJP) utilized in this research was obtained from Xinxiang Fumin Agricultural Technology Co., Ltd. (Xinxiang, China). The raw material was derived from mature jujubes (*Ziziphus jujuba* Mill.), which was subjected to puffing processing technology, including heat (130 °C) and pressure (0.5 MPa, 7 min) treatments, to obtain a porous structure (0.1–0.3 g/cm^3^). The detailed nutritional composition of this PJP batch is presented in Supplementary Table S1, encompassing proximate analysis (moisture, crude protein, crude fat, crude fiber, ash, and carbohydrates) and key bioactive components (flavonoids, polysaccharides, vitamins, etc.), which were determined through standardized analytical methodologies.

The goats were randomly allocated into three groups: a 10% PJP group (basal diet + 10% PJP replacement corn), a 20% PJP group (basal diet + 20% PJP replacement corn), and a control group (basal diet). The diet composition and nutritional levels on a dry matter basis are presented in Table 1. During the 10-day acclimation period, all goats were dewormed with ivermectin and fed free-feeding while adjusting feed intake to ensure that residual feed intake was less than 5% of total supply. At the end of the acclimatization period, fasting weights were recorded for two successive days. The formal feeding trial was conducted for 90 days, with feed intake monitored daily and body weight measured weekly.

### 2.3. Growth Performance

Daily feed intake and residual feed intake were documented for each goat. Total feed intake was quantified as the total feed provision minus the total residual feed intake collected throughout the trial period. The Average daily feed intake (ADFI, g/d) was calculated by dividing the total feed intake by the number of experimental days, with residual feed intake amounts recorded individually for each goat:

(1) $$\mathrm{ADFI}\;(g/d)=\frac{\mathrm{Total}\mathrm{feed}\mathrm{intake}\;(g)}{\mathrm{Experimental}\mathrm{duration}\;(d)}$$

Body weights were measured following a 12 h fasting period. Initial body weight was defined as the mean of two consecutive fasting weights recorded prior to the trial, whereas final body weight was calculated from two successive fasting measurements at the conclusion of the trial. The average daily gain (ADG, g/d) was derived as follows:

(2) $$\mathrm{ADG}\;(g/d)=\frac{\mathrm{Final}\mathrm{body}\mathrm{weight}\;(g)-\mathrm{Initial}\mathrm{body}\mathrm{weight}\;(g)}{\mathrm{Experimental}\mathrm{duration}\;(d)}$$

### 2.4. Determination of Apparent Digestibility

The apparent digestibility (AD) coefficients were determined using the endogenous indicator method, specifically acid-insoluble ash (AIA), following the procedures described by [3]. To ensure metabolic adaptation, the experimental period was set to 48 to 52 days. Fresh fecal samples from HB goats within each experimental group were collected twice daily (morning and afternoon) over five consecutive days. Each daily fecal sample (100 g) was divided into two subsamples: Subsample A (50 g) was immediately homogenized with 10% (*v*/*v*) hydrochloric acid to stabilize nitrogenous compounds and stored at −20 °C pending analysis. Subsample B (50 g) was used to determine initial moisture content before drying at 65 °C to constant weight. The dried samples were pulverized using a 1-mm sieve and stored in airtight polyethylene bags. Following AOAC (2020) protocols, feed and fecal samples were analyzed for AIA, crude protein (CP), ether extract (EE), dry matter (DM), phosphorus (P), calcium (Ca), and crude fiber (CF). Acid detergent fiber (ADF) and neutral detergent fiber (NDF) in feed samples were determined according to [3]. Feed samples were homogenized by quartering, pulverized with a cyclone mill (FOSS Cyclotec™ 1093, Höganäs, Sweden), and stored under desiccation. Nutrient AD was calculated using the formula:

(3) $$AD\;(\%)=(\frac{Nutrient\;intake-Fecal\;nutrient\;output}{Nutrient\;intake})\times 100$$

### 2.5. Slaughter Procedures and Carcass Evaluation

Upon experimental completion, trial goats were batch-slaughtered by treatment group. After a 24 h fast with 2 h water deprivation, live weight was measured pre-slaughter. Exsanguination was performed via cervical severance. Subsequently, head, hooves, and tail were removed; skinning and evisceration of abdominal and thoracic contents followed; and carcass weight was recorded. Dressing percentage was computed. The carcass was dissected into lean meat and bone, with the weights of meat and bone accurately measured. Data were applied to calculate dressing percentage = (carcass weight/live weight) × 100%; meat percentage = (lean meat weight/live weight) × 100%; carcass meat yield = (lean meat weight/carcass weight) × 100%; meat-to-bone ratio = (lean meat weight/bone weight) × 100%.

### 2.6. Serum Antioxidants

On the morning of the final experimental day, prior to morning feeding, jugular venous blood samples were collected from all goats using a dual collection system: 10 mL plain vacuum tubes for serum separation and 5 mL heparin sodium-anticoagulated tubes for whole blood analysis. Serum aliquots were separated via immediate centrifugation at 3500× *g* for 15 min at 4 °C and cryopreserved at −80 °C in sterile polypropylene vials for subsequent biochemical assessment. Oxidative stress parameters, including total antioxidant capacity (T-AOC), glutathione peroxidase (GSH-PX) activity, malondialdehyde (MDA) concentration, superoxide dismutase (SOD) activity, and catalase (CAT) activity, were quantitatively analyzed using standardized commercial kits (Nanjing Jiancheng Bioengineering Institute, Nanjing, China) and established enzymatic assays in accordance with the manufacturer’s protocols (Kit numbers are detailed in the Supplementary Table S2).

### 2.7. Meat Quality

After slaughter, latissimus dorsi muscle samples were harvested from HB goats and processed into air-dried specimens for compositional analysis. Dried samples were analyzed using standardized methods: moisture content (DM) was determined according to Shi et al., [3], crude protein (CP) was measured via the Kjeldahl method; ether extract (EE) was analyzed by Soxhlet extraction; ash content was determined through incineration; and amino acid profiles were analyzed using a Hitachi 835-50 analyzer (Tokyo, Japan). Muscle quality parameters (shear force, water-holding capacity, pH, cooking yield) were evaluated following methodology from a previous study [2], employing standardized instrumental measurements and protocols.

### 2.8. Statistical Analyses

Data were organized using Microsoft Excel 2019 (Microsoft Corp., Redmond, WA, USA) and statistically analyzed using IBM SPSS Statistics 25.0 (IBM Corp., Armonk, NY, USA). Prior to analysis, the normality of data distribution was verified using the Shapiro-Wilk test, and homogeneity of variances was assessed via Levene’s test. One-way analysis of variance (ANOVA) was performed to evaluate differences among multiple groups. For post hoc pairwise comparisons, Duncan’s multiple range test was employed. All statistical tests were two-tailed, with a significance threshold set at *p* < 0.05. Quantitative data are presented as mean ± standard error (SE).

## 3. Results

### 3.1. PJP on the Growth Performance and Apparent Digestibility of HB Goats

Table 2 reveals that no apparent difference was found in the initial weight measurements of all experimental groups (*p* > 0.05), which was in line with the stipulated requirements of the experiment. Compared with the control group, significant enhancements were shown (*p* < 0.05) in the feed-to-gain ratio (F/G), feed intake, and average daily gain (ADG) in both the 10% and 20% PJP substitution groups. Specifically, as the levels of PJP supplementation increased, a progressive increase was observed in both ADG and feed intake. Moreover, it should be noted that Table 3 did not indicate any observed differences (*p* > 0.05) in the digestibility of CP, EE, ASH, NDF, or ADF among the various study groups. However, CP, EE, ASH, NDF, and ADF were significantly higher in the 10% replacement group compared with the control group.

### 3.2. PJP on the Slaughter Performance of HB Goats

As demonstrated in Table 4, there was a significant difference in live weight among the groups (*p* = 0.04). However, no significant differences were detected in the meat-to-bone ratio (*p* > 0.05). Carcass weight, muscle weight, dressing percentage, and meat percentage were observed to increase as the proportion of PJP substitution groups, with significant differences noted (*p* < 0.05). Bone weight, on the other hand, was seen to show a decreasing trend with the increase in PJP substitution groups, though the difference was not significant. Numerous indicators, such as live weight, carcass weight, dressing percentage, and meat percentage, showed no significant variations between the groups with 10% and 20% PJP substitution groups.

### 3.3. PJP on Serum Antioxidant Capacity of HB Goats

As shown in Table 5, no differences in the serum levels of T-AOC, GSH-Px, or MDA were observed among the groups (*p* > 0.05). However, significant differences in the activities of SOD and CAT were detected (*p* < 0.05). When compared with the control group, the levels of T-AOC in the PJP substitution groups first increased and then decreased. The levels of MDA were observed to first decrease and then increase with higher supplementation of PJP; however, all PJP substitution groups maintained lower MDA content compared with the control group. Conversely, the activities of CAT and SOD were observed to progressively increase with elevated levels of PJP.

### 3.4. PJP on the Meat Quality of HB Goats

As presented in Table 6, no significant differences (*p* > 0.05) were observed in DM, CP, EE, or ash content between the 10% and 20% PJP substitution groups and the control group. As shown in Table 7, no remarkable differences (*p* > 0.05) were found in pH at 0 h, pH at 24 h, and water-holding capacity (WHC) between the control group and the PJP substitution groups (10% and 20%). Nevertheless, significant differences were observed in cooking yield and shear force losses (*p* < 0.05). Notably, the meat texture characteristics of the groups with PJP substitution exhibited marked improvement compared with the control group, particularly in the 20% PJP substitution group.

## 4. Discussion

This study evaluated two inclusion levels of puffed jujube powder (PJP), wherein 10% and 20% of corn in the basal diet of Hainan Black (HB) goats was substituted. These levels were specifically selected based on prior research demonstrating that jujube byproducts can effectively replace 15–30% of conventional energy sources in ruminant rations without compromising palatability or nutrient utilization [18,19]. Throughout the experimental period, a clear and consistent dose-dependent relationship was observed. Specifically, the group receiving a 20% PJP inclusion level consistently demonstrated superior performance metrics across multiple key growth and production parameters relative to both the control group and the 10% PJP group. Notably, HB goats fed the 20% PJP diet exhibited significantly higher final body weight (22.58 vs. 21.37 kg), greater average daily gain (83.44 vs. 71.00 g/d), and increased dry matter intake (713.10 vs. 699.30 g/d) compared with those in the 10% PJP group (Table 2). Furthermore, enhancements in key carcass characteristics—such as increased carcass weight, improved dressing percentage, and greater meat yield—along with significant reductions in Warner–Bratzler shear force, indicative of enhanced meat tenderness, were most markedly observed at the 20% supplementation concentration (Table 4 and Table 7). The significantly enhanced performance observed at the higher inclusion level is likely attributable to an increased delivery of bioactive compounds inherent in PJP, including polysaccharides and flavonoids [20,21,22]. These compounds are extensively documented to improve nutrient utilization efficiency, modulate rumen fermentation, and enhance overall metabolic function in ruminants [23,24]. While both PJP supplementation levels elicited improvements in feed conversion ratio and cooking yield relative to the control group, the 20% substitution level elicited more pronounced and significant enhancements in optimizing the key production parameters evaluated under the experimental conditions. This indicates a more favorable economic and production outcome.

The nutritional significance of PJP in ruminant diets is predicated upon its phytochemical constituents, which elicit metabolic adaptations that enhance nutrient utilization efficiency [25,26]. PJP incorporates bioactive constituents including arabinogalactan polysaccharides, flavonoid glycosides, and triterpenic acids, which regulate digestive physiology through the modulation of essential physiological mechanisms [23,27,28]. Specifically, jujube polysaccharides selectively stimulated the proliferation of key fibrolytic rumen bacteria (*Fibrobacter succinogenes* and *Ruminococcus flavefaciens*), as demonstrated by comprehensive 16S rRNA gene sequencing analyses in our unpublished study. Such microbial modulation enhanced the hydrolysis of recalcitrant plant cell walls, releasing fermentable substrates including cellulose-derived oligosaccharides, thereby increasing the efficiency of microbial protein synthesis [29,30]. Additionally, PJP’s unique carbohydrate structure is characterized by a high monosaccharide content and a glucose/fructose ratio of approximately 1.2:1, thereby reducing metabolic energy consumption in glycolysis by circumventing complex carbohydrate breakdown pathways [29]. This enhanced metabolic efficiency was demonstrated by a 37.5% increase in average daily gain (ADG) for the 20% PJP dietary group (83.44 ± 3.21 g/d vs. 60.70 ± 2.89 g/d in controls). Moreover, dietary incorporation of 20% PJP yielded significant improvements in final body weight (28.7 ± 1.3 kg vs. 25.2 ± 1.1 kg), ADG, and average daily feed intake (ADFI: 1.32 ± 0.08 kg/d vs. 1.15 ± 0.07 kg/d) among HB goats following the 90-day experimental interval (Table 2). These findings align with prior research indicating that supplementation of dietary jujube through multiple biological pathways consistently increases weight gain in ruminants [26]. While this study did not explicitly investigate the molecular mechanisms underlying the observed increases in feed intake and average daily gain (ADG), these improvements are potentially attributable to three primary factors: the high concentration of readily digestible carbohydrates in PJP providing immediate energy substrates [6,8]; its enhanced palatability, mediated by volatile organic compounds—an established mechanism for stimulating voluntary feed intake [9,23]; and bioactive constituents, such as polysaccharides and flavonoids [18,19,20], potentially exerting modulatory effects on metabolism. These bioactive components may regulate ruminal fermentation kinetics by stabilizing pH, enhancing enzymatic activity, or influencing specific metabolic pathways such as the AMPK signaling cascade, thereby improving nutrient utilization and protein synthesis efficiency [21,24]. Notably, dietary inclusion of 10% or 20% PJP did not significantly alter digestibility coefficients for crude protein (CP: 68.3 ± 2.1% vs. control 67.8 ± 1.9%), ether extract (EE: 74.5 ± 3.2% vs. control 73.8 ± 2.7%), ash (42.1 ± 1.8% vs. control 41.3 ± 1.7%), NDF, or acid detergent fiber (ADF: 52.4 ± 2.3% vs. control 51.7 ± 2.1%) relative to the control diet (Table 3). This observation indicated that the enhanced growth performance occurred independently of major alterations in core nutrient digestibility, suggesting alternative mechanisms of action. Moreover, the improved feed conversion ratio (FCR: 5.32 ± 0.21 vs. control 6.18 ± 0.25 in the 10% PJP group; 4.89 ± 0.19 vs. control 6.18 ± 0.25 in the 20% PJP group) observed in both PJP-supplemented groups (Table 2) provides corroborative evidence for efficient nutrient partitioning and utilization underpinning the accelerated growth.

Supplementation with PJP enhanced the serum antioxidant status of HB goats, as evidenced by markedly elevated activities of the primary antioxidant enzymes superoxide dismutase (SOD) and catalase (CAT) in the 20% PJP group compared with the control group. This enhancement of enzymatic antioxidant defenses represents a pivotal observation, with particular significance for ruminants reared in tropical environmental stress conditions, which are known to exacerbate oxidative damage [25,26,27,28]. The observed increases in SOD and CAT activities correlate with the established phytochemical profile of PJP, which is characterized by a high abundance of bioactive compounds, including polysaccharides, flavonoids, and triterpenic acids, recognized for their antioxidative properties [29]. These compounds may exert direct antioxidant effects or function indirectly by upregulating the expression and activity of endogenous antioxidant enzymes, such as SOD and CAT, potentially through modulation of cellular signaling pathways, including the Nrf2-Keap1 and JNK/Akt pathways [30,31]. Although glutathione peroxidase (GSH-Px) activity and total antioxidant capacity (T-AOC) remained unchanged, and malondialdehyde (MDA) levels exhibited a non-significant decreasing trend in the PJP substitution group, the significant upregulation of superoxide dismutase (SOD) and catalase (CAT) indicates enhanced systemic antioxidant capacity in goats receiving 20% PJP supplementation. This enhancement is mediated by polyphenolic compounds present in PJP, which activate antioxidant enzymes through modulation of JNK and Akt phosphorylation pathways, consequently reducing cellular reactive oxygen species (ROS) levels and mitigating ROS-induced adverse effects [31]. Consistent evidence confirmed that antioxidant capacity was enhanced by jujube polysaccharides and flavonoids across livestock species. In poultry [32,33,34], SOD and CAT activity were elevated in serum and muscle tissues by these compounds, while MDA levels indicative of lipid peroxidation were reduced, thereby improving meat shelf life. Increased serum T-AOC and GSH-Px activity were demonstrated in bovine studies [35,36,37], alongside improved rumen function and reduced disease incidence. Caprine trials [38,39,40,41] revealed that SOD activity was heightened, MDA concentration was reduced, carcass yield was improved, and meat shear force was diminished. Mechanistically, these effects were mediated through conserved pathways (Nrf2/Keap1, JNK/Akt), which upregulated endogenous antioxidant defenses. The efficacy of jujube bioactives in mitigating oxidative stress and enhancing production outcomes was robustly supported by this multispecies validation.

In this study, meat quality indicators were characterized. Proximate composition analysis of the *Longissimus dorsi* muscle, encompassing dry matter (DM), crude protein (CP), ether extract (EE), and ash content, revealed no significant differences across treatment groups, indicating that PJP supplementation did not alter fundamental nutritional constituents. However, significant improvements in key physical properties were observed in the PJP substitution group. Cooking yield was significantly increased compared with the control, reaching an optimal level at 20% PJP inclusion with an 8.9% increase observed over the control. This improvement suggested that moisture retention was improved during meat thermal processing, weight loss was effectively reduced, and the nutritional capacity of PJP was preserved [42,43]. Correspondingly, shear force values exhibited a significant reduction in PJP-supplemented groups, corresponding to a 12.9% decrease at the 20% supplementation level relative to the control group. This finding indicates a substantial enhancement in meat tenderness. These physicochemical improvements were primarily attributed to PJP-induced elevations in SOD and CAT activities within the muscle tissue, thereby augmenting the overall antioxidant capacity [44,45]. This observation indicated that bioactive antioxidant compounds present in PJP could effectively enhance meat quality. This mechanism potentially reduces oxidative damage to post-mortem muscle proteins and preserves the structural integrity and water holding capacity (WHC) of muscle fibers [41]. Supporting evidence from NB goat results indicates that muscle pH was significantly increased within 24 h post-slaughter, and WHC was enhanced relative to controls through PJP supplementation. This further corroborates that functional additives can regulate the decline in muscle pH, mitigate protein denaturation, and improve meat quality attributes [46]. Previous research on goats has demonstrated that dietary supplementation of plant extracts enhances antioxidant capacity and improves meat quality [47,48,49]. The results demonstrate that key nutritional components were preserved by additives such as PJP, while the physical quality of HB goat meat was significantly improved through antioxidative mechanisms, demonstrating substantial application value and potential in meat production and processing.

In summary, dietary inclusion of 20% PJP significantly enhanced growth performance, carcass yield, antioxidant capacity, and meat quality in HB goats. It effectively optimizes nutrient utilization through bioactive compounds and mitigates oxidative stress in tropical environments. However, several notable limitations were identified in this study. The investigation was limited exclusively to male goats over a 90-day period, which precluded assessment of female-specific effects and long-term impacts. The proposed mechanisms remained hypothetical without empirical validation, as critical measurements of bioactive compound absorption, rumen parameters, and molecular pathway activities were not performed. Sensory evaluations were omitted entirely, and the economic feasibility of incorporating 20% PJP necessitated validation at a commercial scale. Future studies should rigorously validate rumen microbial shifts and comprehensively assess large-scale economic viability.

## 5. Conclusions

This study revealed that incorporating PJP into the diet of HB goats induced significant changes in multiple performance indices. Growth performance parameters, including daily weight gain and feed efficiency, were positively affected, while slaughter performance indicators such as carcass weight and meat yield were also improved. Serum analysis showed that antioxidant capacity was enhanced, as demonstrated by increased activities of SOD and GSH-Px. In terms of meat quality, the dietary addition of PJP improved the apparent digestibility of nutrients, leading to better retention of crude protein. The physical properties of the longissimus dorsi muscle, such as WHC and pH stability, were optimized, which might enhance the juiciness and tenderness of meat. These results demonstrate that PJP supplementation, particularly at the 20% concentration, effectively optimizes nutrient utilization efficiency and mitigates oxidative stress in goats. Although these findings are promising for the application of PJP in sustainable livestock production, further research is required to fully understand its long-term effects and economic feasibility in different farming scenarios.

## Figures and Tables

**Table 1:** Diet composition and nutritional levels (dry matter basis).

Item		Control Group	10% PJP ^(1)^	20% PJP
Composition (%)	King grass	38.00	38.00	38.00
	Alfalfa hay	15.00	15.00	15.00
	Soybean meal	5.00	5.00	5.00
	Palm kernel meal	3.00	3.00	3.00
	Corn	37.50	27.50	17.50
	Puffed jujube powder	0.00	10.00	20.00
	Salt	0.80	0.80	0.80
	Baking soda	0.20	0.20	0.20
	Premix ^(2)^	0.50	0.50	0.50
Nutritional level	Digestible energy (MJ·kg^−1^) ^(3)^	11.54	10.89	10.24
	Crude protein (%)	10.70	10.65	10.50
	Neutral detergent fiber (%)	38.40	39.30	40.10
	Acid detergent fiber (%)	23.30	23.70	24.20
	Calcium (%)	0.45	0.45	0.46
	Available phosphorus (%)	0.32	0.33	0.32

Notes: ^(1)^ Puffed jujube powder. ^(2)^ The premix provides per kg of diet: MnSO_4_ 143 mg, ZnSO_4_ 176 mg, FeSO_4_ 115 mg, CoCl_2_ 6.25 mg, KIO_3_ 20 mg, CuSO_4_ 28 mg, NaSeO_3_ 4 mg, Vitamin A 15.28 mg, Vitamin E 0.47 mg. ^(3)^ Digestible energy is a calculated value; all other values are measured values.

**Table 2:** Effects of puffed jujube powder on growth performance of Hainan Black goat.

Item	Control Group	10% PJP ^(1)^	20% PJP	SEM ^(2)^	*p*-Value
Initial weight/kg	15.12	14.98	15.07	1.32	0.31
Final weight/kg	20.45 ^b^	21.37 ^a^	22.58 ^a^	1.13	0.04
Weight gain/kg	5.33 ^b^	6.39 ^a^	7.51 ^a^	1.27	0.03
Average daily gain/g	59.22 ^b^	71.00 ^a^	83.44 ^a^	2.78	0.04
Average daily feed intake/(g·d^−1^)	498.20 ^b^	699.30 ^a^	713.10 ^a^	4.67	0.05
Feed conversion ratio/%	8.41 ^b^	8.84 ^a^	8.55 ^a^	1.01	0.03

Notes: ^(1)^ PJP = Puffed jujube powder. ^(2)^ SEM = standard error of the mean. Values within the same row bearing no superscripts or the same letter superscripts indicate no significant difference (*p* > 0.05), while different lowercase letters indicate significant differences (*p* < 0.05).

**Table 3:** Effects of Puffed jujube powder on apparent digestibility in Hainan black goat.

Item/%	Control Group	10% PJP ^(1)^	20% PJP	SEM ^(2)^	*p*-Value
Crude Protein (CP)	67.20	73.78	69.65	1.78	0.21
Ether Extract (EE)	87.67	89.31	86.50	2.02	0.25
Ash Content (ASH)	30.31	45.29	35.32	0.89	0.09
Neutral Detergent Fiber (NDF)	26.09	51.82	48.59	0.77	0.15
Acid Detergent Fiber (ADF)	18.54	47.37	44.10	1.01	0.06

Notes: ^(1)^ PJP = Puffed jujube powder. ^(2)^ SEM = standard error of the mean.

**Table 4:** Effects of puffed jujube powder on slaughter performance in Hainan Black goat.

Item	Control Group	10% PJP ^(1)^	20% PJP	SEM ^(2)^	*p*-Value
Carcass weight/kg	11.79 ± 1.38 ^b^	13.23 ± 1.18 ^a^	13.99 ± 1.22 ^a^	0.78	0.05
Muscle weight/kg	9.59 ± 1.99 ^c^	10.70 ± 1.11 ^b^	11.43 ± 1.42 ^a^	1.25	0.04
Bone weight/kg	2.21 ± 0.11	2.13 ± 0.17	2.06 ± 0.21	0.34	0.08
Dressing percentage/%	37.42 ± 2.71 ^b^	41.54 ± 2.09 ^a^	42.41 ± 2.67 ^a^	0.57	0.05
Meat percentage/%	30.43 ± 1.98 ^b^	33.59 ± 2.09 ^a^	34.65 ± 1.82 ^a^	2.23	0.02
Meat yield percentage/%	77.34 ± 2.54	80.88 ± 2.09	81.70 ± 1.99	2.02	0.06
Meat-to-bone ratio/%	4.76 ± 0.29	4.13 ± 0.23	4.06 ± 0.45	0.12	0.07

Notes: ^(1)^ PJP = Puffed jujube powder. ^(2)^ SEM = standard error of the mean. Values within the same row bearing no superscripts or the same letter superscripts indicate no significant difference (*p* > 0.05), while different lowercase letters indicate significant differences (*p* < 0.05).

**Table 5:** Effects of puffed jujube powder on serum antioxidant parameters in Hainan Black goat.

Item/%	Control Group	10% PJP ^(1)^	20% PJP	SEM ^(2)^	*p*-Value
T-AOC (U/mL)	5.98 ± 0.61	6.77 ± 0.78	6.52 ± 0.34	0.23	0.07
GSH-PX (U/mL)	61.21 ± 1.61	65.88 ± 1.89	66.04 ± 2.06	2.87	0.21
MDA (nmol/mL)	4.63 ± 0.32	4.09 ± 0.25	4.22 ± 0.46	0.27	0.09
SOD (U/mL)	58.63 ± 1.78 ^b^	62.23 ± 2.01 ^a^	64.28 ± 2.36 ^a^	3.01	0.04
CAT (U/mL)	44.23 ± 2.23 ^b^	49.23 ± 1.88 ^a^	51.49 ± 2.19 ^a^	2.78	0.05

Notes: ^(1)^ PJP = Puffed jujube powder. ^(2)^ SEM = standard error of the mean. Values within the same row bearing no superscripts or the same letter superscripts indicate no significant difference (*p* > 0.05), while different lowercase letters indicate significant differences (*p* < 0.05).

**Table 6:** Effects of puffed jujube powder on conventional nutrient content in muscle of Hainan Black goat.

Item/%	Control Group	10% PJP ^(1)^	20% PJP	SEM ^(2)^	*p*-Value
Dry Matter (DM)	28.91	27.23	28.09 ± 0.56	2.31	0.76
Crude Protein (CP)	21.77	23.98 ± 1.02	24.11 ± 0.42	1.98	0.06
Ether Extract (EE)	5.34	4.13 ± 0.08	4.43 ± 0.05	0.42	0.12
Ash	1.22	1.17 ± 0.02	1.09 ± 0.01	0.01	0.26

Notes: ^(1)^ PJP = Puffed jujube powder. ^(2)^ SEM = standard error of the mean.

**Table 7:** Effects of puffed jujube powder on muscle quality properties in Hainan Black goat.

Item/%	Control Group	10% PJP ^(1)^	20% PJP	SEM ^(2)^	*p*-Value
pH 0 h	6.54 ± 0.35	6.77 ± 0.28	6.65 ± 0.29	0.37	0.12
pH 24 h	6.21 ± 0.25	6.39 ± 0.33	6.40 ± 0.28	1.23	0.09
Water holding capacity (%)	31.45 ± 2.89	33.23 ± 3.11	32.89 ± 2.56	0.78	0.25
Shear force (N)	24.23 ± 1.89 ^a^	22.22 ± 2.34 ^b^	21.09 ± 2.06 ^c^	2.36	0.02
Cooking yield (%)	60.12 ± 5.56 ^c^	63.23 ± 4.89 ^b^	66.02 ± 6.06 ^a^	5.72	0.03

Notes: ^(1)^ PJP = Puffed jujube powder. ^(2)^ SEM = standard error of the mean. Values within the same row bearing no superscripts or the same letter superscripts indicate no significant difference (*p* > 0.05), while different lowercase letters indicate significant differences (*p* < 0.05).

## Data Availability

Datasets are available upon reasonable request from the authors.

## Supplementary Materials

The following supporting information can be downloaded at https://www.mdpi.com/article/10.3390/ani15152306/s1. Table S1: Nutritional composition analysis of puffed jujube powder; Table S2: Antioxidant kit information.

## Abbreviations

The following abbreviations are used in this manuscript:

ADG	Average daily gain
ADFI	Average daily feed intake
ADF	Acid detergent fiber
AD	Apparent digestibility
AIA	Acid-insoluble ash
CP	Crude protein
CF	Crude fat
DM	Dry matter
EE	Ether extract
GSH-PX	Glutathione peroxidase
MDA	Malondialdehyde
PJP	Puffed jujube powder
SOD	Superoxide dismutase
T-AOC	Total antioxidant capacity
