# Supplementary material for: Effects of Dietary Puffed Jujube Powder on Growth Performance, Apparent Digestibility, and Meat Quality of Hainan Black Goats
Source: Animals (Basel). 2025 Aug 6;15(15):2306. doi: 10.3390/ani15152306 (PMC12345422; doi:10.3390/ani15152306)
Supplement: Supplementary file 1 [file animals-15-02306-s001.zip › animals-3727454-supplementary.pdf]

Table S1. Nutritional composition analysis of puffed jujube powder.

Component (%)	Content (w/w, dry basis)
Moisture	6.2 ± 0.3
Crude Protein	9.8 ± 0.5
Crude Fat	2.1 ± 0.2
Crude Fiber	18.5 ± 0.8
Ash	3.4 ± 0.1
Total Carbohydrates	60.0 ± 1.2
**Bioactive Compounds**	**Content**
Flavonoids (as quercetin)	12.5 ± 0.6 mg/g
Polysaccharides	8.3 ± 0.4 g/100g
Vitamin C	28.7 ± 1.3 mg/100g

All values represent the mean ± standard deviation (n = 3). Additionally, the PJP batch (lot number: *PJP202405*) was characterized by high-performance liquid chromatography (HPLC) and Fourier-transform infrared spectroscopy (FTIR), confirming the presence of key phenolic compounds (e.g., gallic acid, chlorogenic acid) and dietary fiber structures.

Table S2. Antioxidant kit information.

Antioxidant	No.
Glutathione peroxidase (GSH-PX)	A-001-3-2
Total superoxide dismutase (T-SOD)	A-015-2-1
Malondialdehyde (MDA)	A-003-1-2
Superoxide dismutase (SOD)	A-005-1-2
Catalase (CAT)	A-007-1-2

All the kits were supplied by the Nanjing Jiancheng Bioengineering Institute Co., Ltd. (Nanjing, China).
